# Sphingolipidoses: expanding the spectrum of α-synucleinopathies

**DOI:** 10.1007/s00702-025-02925-z

**Published:** 2025-04-17

**Authors:** Daniel Erskine, Agnieszka K. Bronowska, Tiago F. Outeiro, Johannes Attems

**Affiliations:** 1 Metabolic Neurodegeneration Laboratory, Newcastle University, Newcastle, UK; 2 Translational and Clinical Research Institute, Newcastle University, Newcastle, UK; 3 Chemistry - School of Natural and Environmental Sciences, Newcastle University, Newcastle, UK; 4 DZNE, Gottingen, Germany; 5 University Medical Center Gottingen, Newcastle, Germany

**Keywords:** Sphingolipid, Alpha-synuclein, Lysosomal storage disorder, Parkinson’s, Lewy, Neurodegeneration

## Abstract

Although α-synuclein pathology is typically associated with Lewy body diseases and multiple systems atrophy, increasing evidence indicates that it also occurs in a group of lysosomal storage disorders termed sphingolipidoses caused by the incomplete degradation, and subsequent accumulation, of a class of lipids termed sphingolipids. Notably, a number of genes that cause sphingolipidoses are also risk genes for Lewy body diseases, suggesting aetiological links between these distinct disorders. In the present review, we discuss the sphingolipidoses in which α-synuclein pathology has been reported: Gaucher disease, Krabbe disease, metachromatic leukodystrophy, Tay-Sachs disease and Anderson-Fabry disease, and describe the characteristic clinical and pathological features of these disorders, in addition to the evidence suggesting α-synuclein pathology occurs in these disorders. Finally, we evaluate the pathological mechanisms that underlie these rare disorders, with particular attention to how the enzymatic deficiency, substrate accumulation, or both, could contribute to the genesis of α-synuclein pathology and the implications of this for Lewy body diseases.

## Introduction

α-synuclein is a 140 amino acid protein that is abundantly expressed in central nervous system tissue, and also in blood (in erythrocytes and platelets). For reasons not fully known, α-synuclein aggregates into increasingly structured intracellular inclusions that are thought to play a critical role in the group of disorders termed “α-synucleinopathies”. α-synucleinopathies are classified into the Lewy body diseases, such as Parkinson’s disease (PD) and dementia with Lewy bodies (DLB), and multiple systems atrophy (MSA) (Outeiro et al. 2019).

Lewy body diseases (LBDs) are characterised by the accumulation of the protein α-synuclein into intracellular deposits termed Lewy bodies (Koga et al. 2021). Lewy body disease is an umbrella term for the group of diseases in which Lewy bodies are observed, and include: DLB, a cognitive disorder in which parkinsonism can develop contemporaneous or subsequent to cognitive symptoms, respectively; PD, a movement disorder, and PD dementia (PDD), a neurocognitive disorder which occurs in the context of established PD (McKeith et al., 2017). Collectively, LBDs are the second most common neurodegenerative disorder after Alzheimer’s disease, with PD alone occurring in 1,087 per 100,000 individuals aged 70–79 years old (Heidebrink 2002; Pringsheim et al. 2014). Lewy bodies are the defining pathological feature of PD, PDD and DLB, though their role in the disease process remains unclear and their abundance and distribution does not appear to be associated with phenotypic severity (Gomez-Tortosa et al. 1999; Martin et al. 2023). Nevertheless, irrespective of the association between Lewy bodies and clinical decline in LBDs the protein α-synuclein is thought to play a prominent role in the disease process due to variants and duplications in the α-synuclein gene, *SNCA*, increasing risk of LBDs, and reduced risk of LBD in those taking drugs that pharmacologically reduce α-synuclein transcription (Byers et al. 2011; Campelo and Silva 2017; Mittal et al. 2017). Therefore, despite an uncertain role for Lewy bodies in LBDs there is compelling evidence to support a central role for α-synuclein dysregulation and, therefore, there is considerable interest in identifying determinants of α-synuclein dysregulation that could be amenable to therapeutic intervention.

MSA is the only known synucleinopathy that is characterised by the accumulation of α-synuclein within oligodendroglia known as GCIs, or Papp-Lantos bodies (Jellinger and Lantos 2010), but neuronal cytoplasmic inclusions (NCIs) are seen as well. In contrast to LBDs, MSA occurs in approximately 113 per 100,000 individuals over 75 years old (Kaplan et al. 2023). Clinically, MSA presents with a heterogeneous phenotype and several sub-types, including a parkinsonian motor sub-type and a ataxia-predominant cerebellar sub-type, though autonomic dysfunction is found in a significant proportion of cases irrespective of sub-type (Stankovic et al. 2023). Recently, the oligodendroglial hypothesis of MSA has been challenged by studies indicating neuronal α-synuclein pathology (i.e., NCIs) could drive the clinical decline that characterises the disorder (Wiseman et al. 2025). However, as with Lewy body diseases, the underlying cause of MSA remains unclear though α-synuclein accumulation has long been thought to be an important contributor to the neurodegeneration that characterises MSA and, therefore, a major target for candidate therapeutics.

Although α-synuclein has typically been thought of in the context of Lewy body diseases and MSA, it is becoming increasingly clear that its accumulation is associated with other, typically rarer, conditions. It is notable that the accumulation of α-synuclein in such rare disorders is not always associated with histologically manifest inclusions bodies as observed in Lewy body diseases and MSA; however, in the present article we will use a broad interpretation of “α-synucleinopathy” that includes disorders in which it accumulates into inclusions or has prion-like capacity on aggregation assay. We feel our broad interpretation is important as the role of α-synuclein in the described disorders remains to be elucidated. Nevertheless, the observation of changes to α-synuclein reminiscent of those in Lewy body diseases and MSA is important as the typically monogenic aetiology of these disorders means they may provide critical insights into the factors that govern α-synuclein aggregation. Furthermore, many of the rare diseases in question have a relentless progression and limited therapeutic options; thus, identifying the involvement of a process associated with Lewy body diseases and MSA, common diseases in which significant drug discovery is on-going, could help with repurposing future therapies for these otherwise fatal conditions.

In the present article, we will discuss the sphingolipidoses, a group of rare diseases in which α-synuclein aggregation has been identified, and discuss the potential relevance of these findings for LBDs, MSA, and sphingolipidoses.

## Sphingolipidoses

Sphingolipidoses are a group of lysosomal storage disorders typically resulting from bi-allelic loss-of-function variants in lysosomal enzymes responsible for degrading sphingolipids, a group of lipids associated with cell membranes and lipids, and which are enriched in the central nervous system (Abed Rabbo et al. 2021). In most sphingolipidoses, reduced enzyme function leads to the accumulation of sphingolipid substrates to toxic levels, though some result from dysfunction of catalytic enzymes or other essential co-factors for sphingolipid catabolism. It is notable that a number of risk genes for Lewy body diseases, though not MSA, encode lysosomal enzymes which in a bi-allelic state underlie sphingolipidoses, suggesting an association between these metabolic pathways and α-synucleinopathy, particularly Lewy body diseases (Erskine et al. 2021).

Sphingolipids are a class of bioactive lipids with key roles in the cell cycle, cell death, senescence, metabolism and inflammation (Hannun and Obeid 2018). In the context of α-synuclein accumulation, sphingolipids are potentially highly relevant as they are enriched within lipid rafts, protein-rich areas of membranes in which α-synuclein is also located, and thus changes in the balance of distinct sphingolipid species may alter the localisation or solubility of α-synuclein in lipid rafts (Bieberich 2018). Furthermore, sphingolipids have intrinsic properties, such as the propensity to cluster, which may be relevant in the context of protein aggregation, particularly if sphingolipids have the capacity to cross-seed or directly interact with α-synuclein (Sonnino et al. 2006). The role of sphingolipids in α-synuclein aggregation is suggested by the over-representation of genes encoding enzymes responsible for the degradation of sphingolipids amongst risk genes for Lewy body diseases, particularly PD (Lin et al. 2019).

### Gaucher disease

Gaucher disease is caused by bi-allelic loss-of-function variants in *GBA1*, which encodes the lysosomal glucocerebrosidase that hydrolyses glucosylceramide into glucose and ceramide (Stirnemann et al. 2017). Loss-of-function of glucocerebrosidase leads to the accumulation of glucosylceramide and a de-acylated derivative, glucosylsphingosine, of which the latter is formed by cleavage of a fatty acyl chain in glucosylceramide by acid ceramidase (Mistry et al. 2014). Dysfunction of *GBA1* and/or the accumulation of its substrates underlie three distinct disease sub-types characterised on the basis of their clinical presentation.

Type 1, or non-neuronopathic Gaucher disease, is the most common in the western world, accounting for approximately 94% of all cases and is especially common in the Ashkenazi Jewish population, where it has a 1 in 850 prevalence compared to 1–2 per 100,000 in the non-Ashkenazi Jewish population (Charrow et al. 2000; Horowitz et al. 1998). Type 1 Gaucher disease has a variable onset and phenotype, though over half of patients report symptom onset prior to the age of 20 years old and, of these, almost half were diagnosed before the age of six years old (Charrow et al. 2000). Common features of Gaucher Type 1 include fatigue, splenomegaly, hepatomegaly, thrombocytopenia and chronic joint and limb pain (Stirnemann et al. 2017). Life expectancy in Gaucher disease Type 1 is variable, and median age at death reported by specific studies ranges from 60 to 68 years (Nalysnyk et al. 2017).

Type 2 and Type 3 Gaucher disease differ from Type 1 Gaucher disease as the central nervous system is pathologically affected, and they are estimated to occur in 1 in 100,000–300,000 births, making them less common than Type 1 (Nalysnyk et al. 2017). Type 2 Gaucher disease typically onsets in infants aged three to six months old with a triad of neurological symptoms that include opisthotonos, bulbar signs (particularly swallowing difficulties) and oculomotor paralysis (Mignot et al. 2006). Psychomotor regression often occurs subsequent to diagnosis in addition to myoclonic seizures and splenomegaly, with death typically occurring before the third year of life (Mignot et al. 2013). Type 3 Gaucher disease exhibits the visceral manifestations of Type 1 Gaucher disease, typically alongside oculomotor dysfunction and, in many cases, variable degrees of cerebellar ataxia and dementia (Kraoua et al. 2011). Type 3 onset is typically between one and three years old with a disease course of three to four decades (Hughes and Pastores 2023).

The characteristic pathological feature of Gaucher disease is the accumulation of glucosylceramide in enlarged macrophages, termed Gaucher cells, which are observed within bone marrow, spleen and liver of Gaucher disease cases (Lee 1968). Due to the minimal neurological involvement of the brain in Type 1 Gaucher disease, there is limited data about its neuropathology; however, one study reported no increase in glucosylceramide in one adult patient with Gaucher disease Type 1 despite deficient glucocerebrosidase activity and accumulated Gaucher cells (Soffer et al. 1980). A subsequent study has reported widespread gliosis in brain tissue from Gaucher Type 1 patients, particularly in the hippocampus, primary visual cortex and pyramidal cell layers of the neocortex (Wong et al. 2004). Type 2 and 3 cases typically exhibit accumulation of glucosylceramide in the occipital cortex, but elevated levels were also observed in frontal, temporal, cerebellum, thalamus and striatum. In Type 2 brains, Gaucher cells and gliosis were observed in regions with abundant glucosylceramide; in contrast, despite an identical pattern of elevated glucosylceramide in Type 3 brains, no Gaucher cells or gliosis was observed (Kaye et al. 1986).

Gaucher disease is of interest in the context of α-synucleinopathy as *GBA1* variants are amongst the most common contributors to risk of developing idiopathic PD (Parlar et al. 2023). Furthermore, parkinsonism has long been recognised to be a feature of some older patients with Gaucher disease (Tayebi et al. 2003). On this basis, neuropathological examinations of Gaucher disease patients have been conducted *post-mortem* to determine whether α-synuclein pathology is present. These studies have reported Lewy body pathology reminiscent of that observed in PD, such as eosinophilic spherical inclusions in the midbrain and Lewy bodies and neurites in the hippocampus, in a sub-set of Gaucher disease cases though, perhaps surprisingly, these were exclusively in Type 1 non-neuronopathic cases, where they occurred in 4/7 cases aged 53–75 years old (Wong et al. 2004). Although astrogliosis was reported in the regions in which Lewy bodies were observed in Gaucher disease, this was also noted in cases without Lewy bodies, and neuronal loss was not reported to accompany Lewy bodies in the regions in which it was evaluated (hippocampus and cerebral cortex) (Wong et al. 2004). Therefore, although Lewy bodies are present in a sub-set of Gaucher disease cases, their association with other pathological features is not clear, though it is notable that all cases with Lewy body pathology had clinical parkinsonism (Wong et al. 2004).

Rodent models of *Gba1* knockout or specific missense variants associated with PD and Type 3 Gaucher disease, indicate that glucocerebrosidase deficiency leads to increased abundance of α-synuclein and a heightened vulnerability to α-synuclein-mediated neurodegeneration (Migdalska-Richards et al. 2017; Kim et al., 2018a, Polinski et al. 2022). As many models of *Gba1* deficiency develop α-synuclein accumulation *de novo*, it seems likely that loss of glucocerebrosidase function due to particular missense variants not only enhances vulnerability to α-synucleinopathy, but could be both necessary and sufficient for the generation of α-synuclein pathology under certain conditions (Polinski et al. 2021; Yun et al. 2018; Sardi et al. 2011). Given the close association between glucocerebrosidase deficiency and α-synucleinopathy, considerable efforts have been undertaken to elucidate their relationship. A simple explanation for the association between *GBA1* and α-synuclein is that, as α-synuclein is degraded in the lysosome and glucocerebrosidase is a lysosomal enzyme, the loss of glucocerebrosidase activity compromises lysosomal function, leading to the amyloid conversion of highly expressed aggregation-prone proteins, such as α-synuclein (Bae et al. 2015; Mazzulli et al. 2011). However, there are a large number of lysosomal storage disorders, almost all of which lead to altered lysosomal activity, and yet very few of such disorders are known to manifest α-synuclein pathology, perhaps suggesting the underlying mechanism is more nuanced. Glucocerebrosidase directly interacts with α-synuclein in the lysosomal pH range, and glucocerebrosidase may interact with α-synuclein, altering its structure to expose more of the protein to lysosomal proteases, together suggesting glucocerebrosidase may be directly involved in the degradation of α-synuclein (Yap et al. 2011, 2015). Consistent with a role for glucocerebrosidase in degrading α-synuclein, studies in cultured neurons indicate activation of glucocerebrosidase reduces α-synuclein aggregation (Mazzulli et al. 2016). Therefore, there is evidence to indicate glucocerebrosidase may directly interact with α-synuclein to promote its degradation, suggesting its loss of function could promote the accumulation of α-synuclein by inhibiting its degradation.

In addition to the potential direct role of diminished glucocerebrosidase activity on α-synuclein aggregation, further studies have indicated that the substrates degraded by glucocerebrosidase could cross-seed α-synuclein. Glucosylceramide is the primary substrate associated with *GBA1* dysfunction though a de-acylated form of this lipid formed by removal of one fatty acyl chain by acid ceramidase, glucosylsphingosine, is also known to accumulate in Gaucher disease (Murugesan et al. 2016). There is growing interest in the role of de-acylated ceramides, so-called lyso-sphingolipids, in the aetiology of sphingolipidoses, in part due to their pro-inflammatory qualities and ability to escape the lysosome (van Eijk et al. 2020). Analyses of the pro-aggregation effects of glucosylceramide and glucosylsphingosine using in vitro α-synuclein aggregation assays indicates glucosylsphingosine is particularly effective at seeding α-synuclein aggregation (Taguchi et al. 2017). Furthermore, inhibition of glucosylsphingosine production via acid ceramidase reduces α-synuclein aggregation in *GBA1* deficient cells, further suggesting that glucosylsphingosine accumulation may be a key driver of α-synuclein aggregation in *GBA1* deficiency (Kim et al. 2018b). It is not clear why glucosylsphingosine may promote the aggregation of α-synuclein though it is notable that it is positively charged, which is unusual for a lipid, and previous studies have indicated that positively charged molecules that interact with α-synuclein can neutralise its negatively-charged C-terminus, inducing conformational changes thought to be permissive to aggregation (Fernandez et al. 2004).

In summary, a sub-population of Gaucher disease patients with Type 1 Gaucher disease manifest Lewy body pathology and rodent models of Gaucher disease develop *de novo* α-synucleinopathy, suggesting a link between the metabolic dysfunction in Gaucher disease and α-synuclein aggregation. There is evidence that glucocerebrosidase may aid the degradation of α-synuclein, suggesting the loss of its function could promote the accumulation and subsequent aggregation of α-synuclein. Furthermore, the lipid substrates that accumulate in Gaucher disease have the capacity to induce the aggregation of α-synuclein, together suggesting that α-synuclein aggregation resulting from glucocerebrosidase dysfunction could reflect the combined effects of decreased turnover of α-synuclein and the accumulation of lipid substrates permissive to aggregation.

### Krabbe disease

Krabbe disease is a typically infantile disorder resulting from bi-allelic loss-of-function variants in the *GALC* gene, which encodes the lysosomal enzyme galactocerebrosidase (Deane et al. 2011). As with glucocerebrosidase in Gaucher disease, the primary function of galactocerebrosidase is in the hydrolysis of the galactose group from galactosylceramide (Reza et al. 2021). However, unlike Gaucher disease, the primary substrate, galactosylceramide, is not thought to underlie the disorder as recent studies have indicated that galactosylsphingosine, a de-acylated derivative of galactosylceramide generated by the removal of a fatty acyl chain by acid ceramidase, which is more commonly termed psychosine (Li et al. 2019). The accumulation of psychosine to toxic levels is thought to underlie several sub-types of Krabbe disease, which are distinguished based on the age at onset of symptoms: infantile (under 36 months), juvenile (36 months to 18 years) and adult (18 years and over). Krabbe disease is rare, with an estimated birth incidence of approximately 1 in 310,000 live births (Ghabash et al. 2022). The worldwide Krabbe registry suggests an incidence of approximately 1 in 250,000 in the USA based on newborn screening of 1,000,000 infants (Duffner et al. 2011).

Most cases of Krabbe disease onset in infancy, approximately 62% in early infancy (within 6 months of birth) and 10% in late infancy (onset 7–36 months) (Duffner et al. 2012). Infantile Krabbe disease cases very often present with persistent irritability and crying, but further features with progression include poor head control, stiffness, loss of vision and seizures (Duffner et al. 2011). Infantile Krabbe disease is relentlessly progressive, and the majority of early infantile cases succumb within 18 months of onset, and late infantile cases typically within 9.5 years of onset (Duffner et al. 2011).

Juvenile-onset Krabbe disease accounts for approximately 22% of all Krabbe disease cases and presents with changes in gait followed by stiffness, poor feeding and loss of developmental milestones (Duffner et al. 2012). The course of juvenile-onset Krabbe disease is variable, in part due to the wide age range of presentation, but the majority of patients succumb within 10 years of the onset of symptoms (Jain and De Jesus 2020). Adult-onset Krabbe disease is the least common of all sub-types, accounting for just under 5% of all cases (Duffner et al. 2012). The rarity of adult-onset Krabbe disease means it is relatively poorly characterised, but case reports indicate difficulty walking, spastic paraparesis and weakness in lower limbs as common features (Satoh et al. 1997; Debs et al. 2013).

**Fig. 1:**
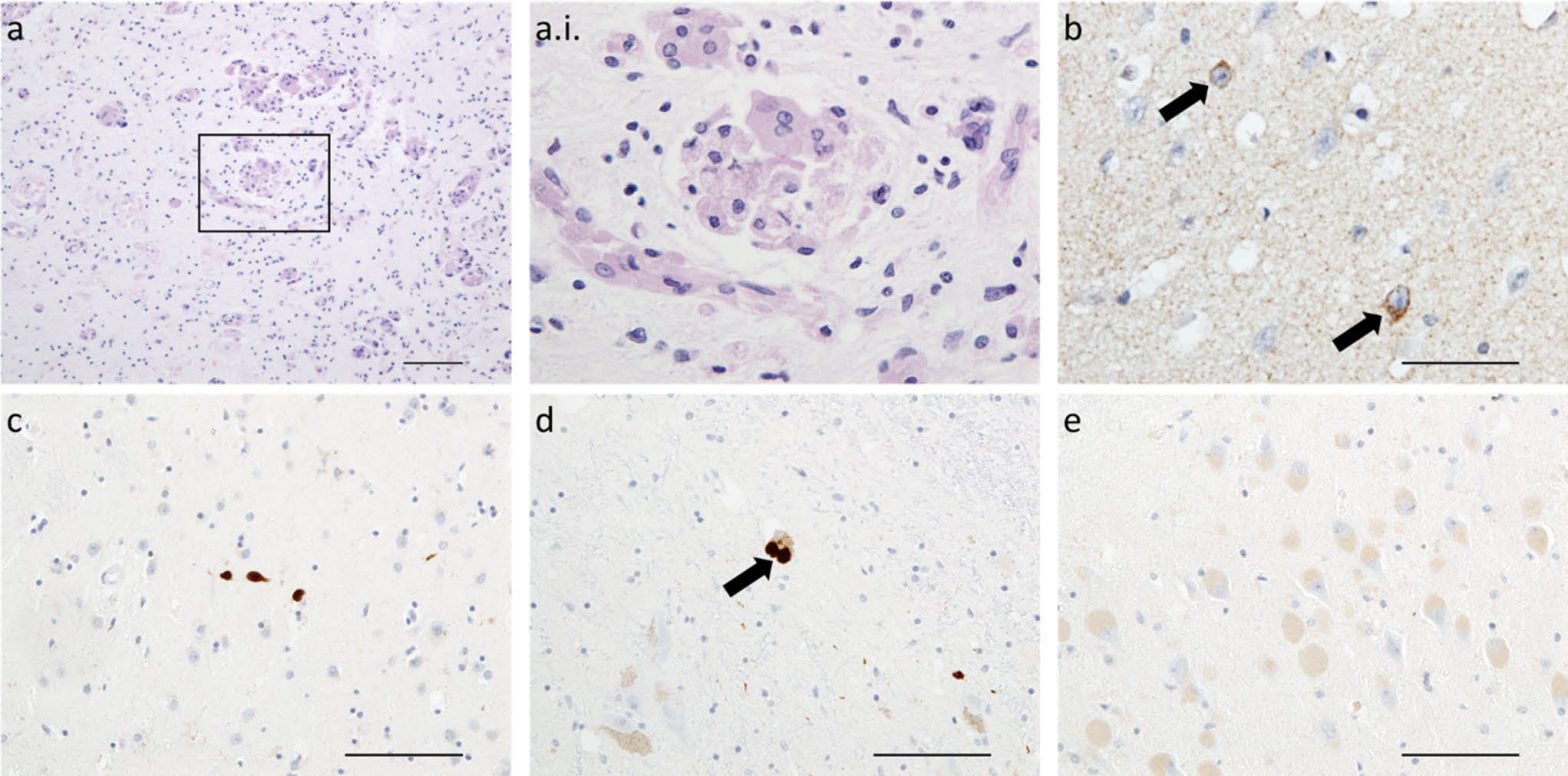
Neuropathology of Krabbe disease and Tay-Sachs disease. Periodic acid Schiff-positive mutli-nucleated globoid cells in areas of demyelination are the neuropathological hallmark of Krabbe disease, as seen here in the brain of a 12 month old who succumbed to Krabbe disease (**a** & a.i.). Although Krabbe disease prominently affects the white matter, intraneuronal α-synuclein accumulations are observed in a sub-population of cortical neurons of the same case (arrows; **b**). In a 62 year old with Tay-Sachs disease, α-synuclein accumulations with some resemblance to Lewy bodies and neurites are observed in the amygdala (**c**) and substantia nigra (**d**), but no accumulation was noted in ballooned neurons in the hippocampus of this case (**e**). Scale bars = 100 μm (**a**, **c**, **d** & **e**), 50 μm (**b**)

The brains of infants and juveniles with Krabbe disease typically exhibit widespread loss of myelin throughout the cortex, deep grey matter and brainstem. The characteristic histological feature of Krabbe disease is the accumulation multi-nucleated PAS-positive macrophage-type cells termed globoid cells, which typically aggregate in perivascular regions of white matter subject to demyelination (Fig. 1a). Demyelination is less extensive in adult Krabbe disease patients, where it is typically restricted to the occipital white matter.

Krabbe disease is of interest in the context of Lewy body diseases as the *GALC* gene has also been associated with risk of PD (Li et al. 2018; Senkevich et al., 2023b). The twitcher mouse, which is considered to be an authentic model of Krabbe disease, manifests α-synuclein aggregates that are labelled by the fibrillar stain, Thioflavin-T (Smith et al. 2014; Abdelkarim et al. 2018). Neuropathological examination of Krabbe disease cases indicates α-synuclein aggregates as diffuse intracellular aggregates rather than Lewy bodies (Fig. 1b), and evaluation of the seeding potential of these cases using seeded aggregation assays demonstrates they contain seed-competent α-synuclein pathology reminiscent of α-synucleinopathies (Hatton et al. 2022; Smith et al. 2014). There is evidence to indicate that α-synuclein aggregation could contribute to Krabbe disease as knockout of α-synuclein leads to a modest, but significant, increased lifespan and attenuated phenotype in Krabbe disease twitcher mice (Abdelkarim et al. 2018).

Unlike with Gaucher disease, there is compelling evidence to indicate that Krabbe disease is primarily caused by the accumulation of a lyso-sphingolipid, in this case psychosine (Li et al. 2019). Psychosine, or galactosylsphingosine, is similar to glucosylsphingosine in Gaucher disease, albeit with a galactose rather than glucose head-group. Nuclear magnetic resonance imaging-based studies have suggested that psychosine can directly interact with the C-terminus of α-synuclein, inducing structural changes that may be permissive to aggregation (Abdelkarim et al. 2018). Therefore, as with Gaucher disease, there is evidence to suggest that lyso-sphingolipids which accumulate in Krabbe disease could interact with α-synuclein and promote its aggregation. It is less clear whether galactocerebrosidase interacts with α-synuclein or has a putative role in degrading α-synuclein, as with glucocerebrosidase.

In summary, α-synuclein pathology has been reported in Krabbe disease rodent models and human *post-mortem* tissue, and there is evidence that the primary toxic substrate to accumulate in Krabbe disease, psychosine, interacts with α-synuclein in a manner that may promote aggregation. However, no study has yet investigated whether galactocerebrosidase has a role in the degradation of α-synuclein, as has been proposed for glucocerebrosidase, and thus it remains unclear as to what drives the aggregation of α-synuclein in Krabbe disease.

### Metachromatic leukodystrophy

Metachromatic leukodystrophy (MLD) is a rare and progressive white matter disorder typically resulting from bi-allelic loss-of-function variants in the *ARSA* gene which encodes arylsulphatase A, a lysosomal enzyme responsible for the de-sulphation of sulphatide into galactosylceramide (Gieselmann 2008). Accumulation of sulphatides throughout the brain, as the result of deficient arylsulphatase A activity, is thought to lead to the progressive loss of white matter, with resulting loss of motor and/or cognitive capacity (Dali et al. 2015). MLD is a rare condition, typically occurring in 0.16–1.85 per 100,000 live births across European, Asian and American populations, with even higher prevalence in Habbanite Jews (1 in 75 births) and Navajo Native Americans (1 in 2,520 births) (Chang et al. 2024; Zlotogora et al. 1980; Holve et al. 2001). As with Krabbe disease, there are a number of sub-types characterised by age of onset, the most common of which is late infantile, followed by juvenile and, more rarely, adult-onset (van Rappard et al. 2015).

Late infantile MLD onsets in the first 30 months of life, with regression of psychomotor function, progressing to frequent spasms, irritability and often seizures (van Rappard et al. 2015). Progression is rapid and affected children typically succumb with five years of disease onset. The juvenile form of MLD onsets between 2.5 and 16 years of age, typically with behavioural and learning problems, developing into spasticity and cognitive impairment, often alongside epilepsy (Gieselmann 2008; van Rappard et al. 2015). Disease duration is highly variable in juvenile-onset MLD, and the terminal stage can last several years. Adult-onset MLD typically presents over the age of 16 years with marked behavioural changes, including memory deficits, emotional instability and psychotic features (Gieselmann 2008). In contrast to earlier-onset sub-types, adult-onset MLD has a more insidious progression and longer course.

*ARSA* has recently been confirmed to be a rare risk gene for Parkinson’s disease (Senkevich et al. 2023a). A previous study in cellular models suggested that arylsuphatase A could act as a chaperone for α-synuclein, the loss of function of which contributes to an increased propensity to protein aggregation (Lee et al. 2019). Neuropathological studies of MLD are rare, but a study of two MLD cases reported histological evidence of α-synuclein pathology in the putamen and cerebral white matter, though as diffuse cytoplasmic inclusions and puncta, rather than Lewy bodies or neurites (Suzuki et al. 2007). We have recently added to these findings by reporting that MLD cases can exhibit diffuse α-synuclein aggregates in vulnerable regions, such as the dentate nucleus, and that α-synuclein elicits a significant response on seeded aggregation assay, suggesting it has seed-competent characteristics reminiscent of those observed in α-synucleinopathies (Ghanem et al. 2024). However, it is not clear the extent to which α-synuclein accumulation associates with neuronal loss, though neuronal loss is not a phenomenon typically observed in MLD.

**Fig. 2:**
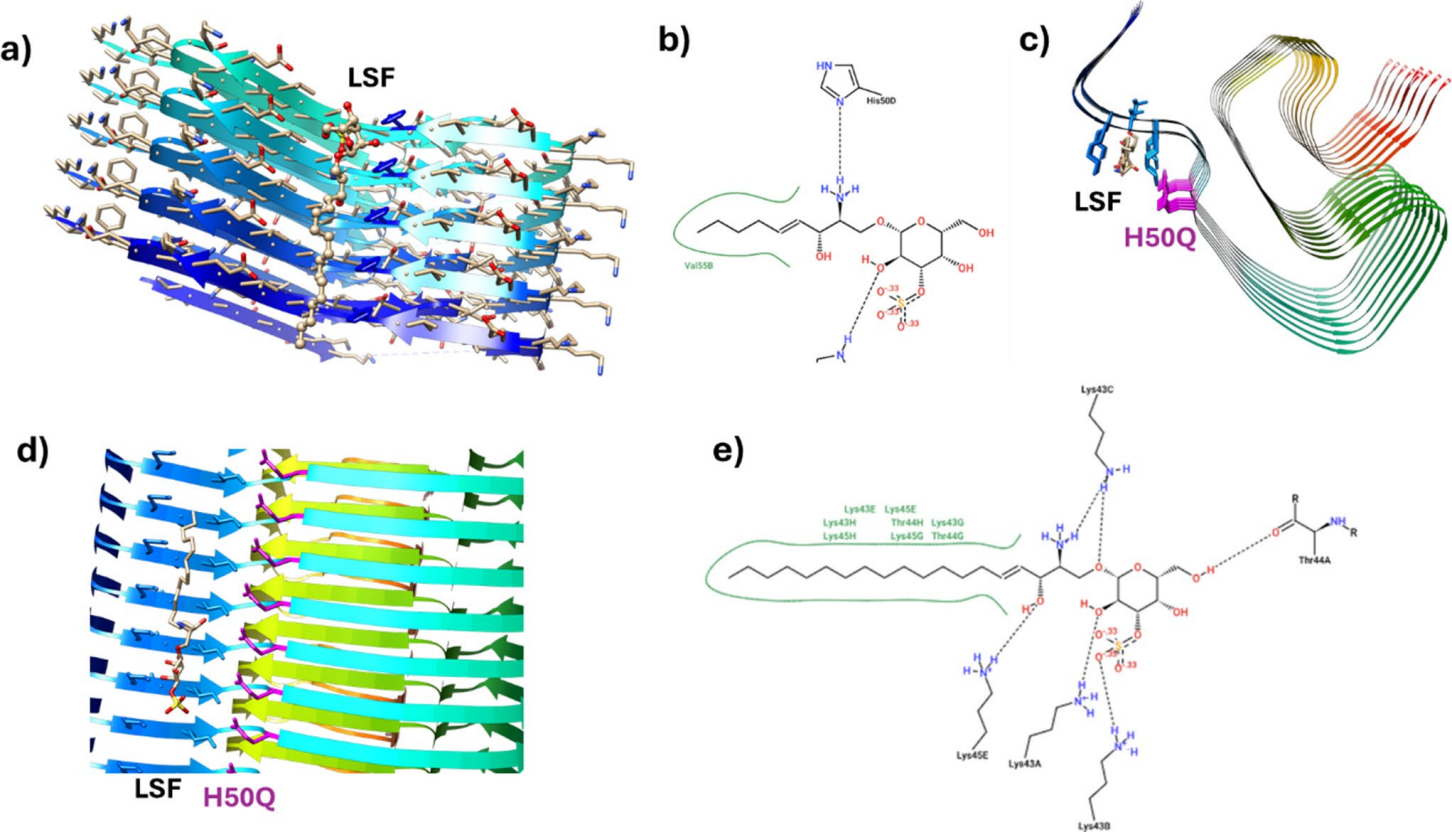
Lyso-sulphatide (LSF) interactions with wild-type α-synuclein. Interactions between lyso-sulphatide and the αSyn fibril (PDB: 8A4L) are illustrated by the docked lyso-sulphatide (LSF). The primary amine group of lyso-sulphatide is involved in favourable interactions with H50, and faces a small cavity comprised of G51 and A53. The sugar is involved in an additional stabilising H-bond, and interactions with the sphingosine chain are stabilised through the classical hydrophobic effect. Histidine H50 in every strand of the fibril is coloured blue (**a**). 2D interaction between lyso-sulphatide and the α-synuclein fibril with residues labelled (**b**). Lyso-sulphatide (LSF) interactions with α-synuclein H50Q missense mutant fibrils (PDB: 6PEO) are shown (**c**-**e**). A view from above (**c**) and the side (**d**) demonstrates lyso-sulphatide interactions with H50Q fibrils. 2D interaction plot between lyso-sulphatide and α-synuclein H50Q fibrils with key residues labelled (**f**)

Although arylsulphatase A has been suggested to be an α-synuclein chaperone, it is not clear if loss of this putative capacity is responsible for the link between *ARSA* and α-synucleinopathy. Although sulphatide is the primary substrate that accumulates in MLD, a de-acylated derivative termed lyso-sulphatide (sulpho-galactosylsphingosine), is also observed in higher abundance in the MLD brain compared to controls (Toda et al. 1990). Lyso-sulphatide, which is the sulphated form of psychosine known to cause Krabbe disease, has been postulated to be a more toxic substrate than sulphatude and could significantly contribute to neurodegeneration and clinical decline in MLD (Blomqvist et al. 2011). Our enhanced-pressure molecular dynamics simulations combined with hotspot mapping and molecular docking indicate that lyso-sulphatide, but not sulphatide, can directly interact with α-synuclein through its amine group, binding to α-synuclein in the region of H50, G51 and A53, which are all residues known to be the location of missense variants associated with PD that enhance α-synuclein aggregation (Fig. 2) (Flagmeier et al. 2016). Some of these binding modes revealed site-specific lysolipid-fibril interactions that are distinct from those reported for sphingolipids (Frieg et al. 2022). Furthermore, these simulations indicated that lyso-sulphatide interactions with α-synuclein are enhanced by the H50Q variant associated with PD, suggesting interactions between α-synuclein and lyso-sulphatide are enhanced by missense variants associated with α-synuclein pathology. Therefore, in addition to diminished putative chaperone activity through arylsulphatase A loss of function, MLD cases may also manifest α-synuclein pathology due to direct interactions between lyso-sulphatide and α-synuclein.

In summary, although there is evidence that arylsulphatase A can act as an α-synuclein chaperone, and thus its dysfunction may result in α-synuclein misfolding, there is also evidence that lyso-sulphatide can interact with α-synuclein in a manner that may promote aggregation. Further studies are warranted to determine the extent to which these effects on α-synuclein may combine to promote α-synuclein aggregation.

### GM2 gangliosidoses

GM2 gangliosidosis is an umbrella term for two disorders, Tay-Sachs disease and Sandhoff disease, which result from impaired degradation of GM2 gangliosides as the result of bi-allelic loss-of-function variants in *HEXA* and *HEXB*, respectively, which encode the subunits of the lysosomal enzyme, hexosaminidase (Leal et al. 2020). Hexosaminidase hydrolyses the N-acetylgalactosamine from GM2 gangliosides to convert them to GM3 gangliosides, the loss of function of which impedes the degradation of GM2 gangliosides and their subsequent accumulation to toxic levels (Cachon-Gonzalez et al. 2018). GM2 gangliosidoses are rare disorders, with estimated prevalences of 1 in 201,000 to 1 in 222,000 for Tay-Sachs disease and 1 in 384,00 to 1 in 422,000 for Sandhoff disease in the general population (Meikle et al. 1999). However, the carrier frequency of *HEXA* variants associated with Tay-Sachs disease is higher in the Ashkenazi Jewish (1 in 30) and eastern Quebec French Canadian (1 in 14), compared to the general population (1 in 300) (Kaback 2000).

As with many similar disorders, GM2 gangliosidoses are differentiated into sub-types based on the age of onset, and Tay-Sachs disease and Sandhoff disease are virtually clinically indistinguishable. The classic form of GM2 gangliosidosis onsets in infancy, typically between 3 and 6 months of age, with an exaggerated startle response and a “cherry red spot” on the retina and developing into a loss of previously acquired milestones, seizures and early mortality, typically within several years of diagnosis (Bley et al. 2011; Xiao et al. 2022). Subacute juvenile GM2 gangliosidoses typically onsets after two years of age with a variable phenotype typically involving gait abnormalities and loss of milestones, developing into seizures and early mortality, typically within the second decade of life (Toro et al. 2020; Xiao et al. 2022). Adult GM2 gangliosidosis is also phenotypically variable but often associated with psychiatric features, weakness of lower limbs, muscle atrophy, abnormal gait and tremor (Toro et al. 2020; Leal et al. 2020; Xiao et al. 2022).

Neuropathologically, GM2 gangliosidoses manifest widespread ballooning of neurons throughout the cerebrum, cerebellum and brainstem, with affected neurons manifesting a nucleus distended by accumulated gangliosides within the cell body and often up-regulation of apoptotic markers (Huang et al. 1997). Notably, rodent models of GM2 gangliosidoses recapitulate the cellular pathology observed in human cases of Tay-Sachs and Sandhoff disease, and also manifest α-synuclein pathology that is ameliorated by expression of functional hexosaminidase, suggesting a direct link between hexosaminidase dysfunction and α-synuclein aggregation (Cachón-González et al. 2014). Notably, crossing Sandhoff disease mice with α-synuclein knockout mice, thus creating Sandhoff disease mice without α-synuclein, demonstrated a reduction in microgliosis alongside improvements in autophagy-lysosomal activity and mitochondrial function, in addition to attenuated dopaminergic neuronal loss (Suzuki et al. 2016). Taken together, these findings suggest α-synuclein accumulation is a feature of rodent models of GM2 gangliosidosis, and it may contribute to pathological changes in these disorders.

There is relatively little human neuropathological data to indicate α-synuclein accumulation is a feature of GM2 gangliosidoses. One study in two Sandhoff and one Tay-Sachs case, all of which were aged three years old or younger at the time of death, suggested punctate α-synuclein immunoreactivity in the neuropil and some labelling of ballooned neurons in all cases (Suzuki et al. 2007; Brekk et al. 2020). We have encountered one case of late onset Tay-Sachs disease, deceased at 62 years old, with evidence of Lewy body pathology in the midbrain and amygdala, but further studies are warranted to demonstrate whether α-synucleinopathy is a feature of GM2 gangliosidoses and the role of hexosaminidase dysfunction and/or GM2 ganglioside accumulation promotes this process (Fig. 1c-d).

In summary, although there is evidence to suggest hexosaminidase activity may be protective against α-synucleinopathy, it is not clear whether GM2 gangliosides interact with α-synuclein or whether this reflects the activity of the enzyme in promoting the degradation of α-synuclein.

### Anderson-Fabry disease

Anderson-Fabry disease is a rare X-linked disorder resulting from variants in the *GLA* gene, which encodes the lysosomal enzyme α-galactosidase A (Duro et al. 2018). α-galactosidase A catalyses the hydrolysis of the terminal α-galactose moiety from a number of glycolipids and polysaccharides, including globotriaosylceramide (Gb3) (Guce et al. 2010). Loss-of-function variants in *GLA* lead to the accumulation of Gb3 and related α-galactosylated glycolipids in multiple organs, including the heart, kidneys, blood vessels and nervous system (Pieroni et al. 2024). Anderson-Fabry disease has a prevalence of 1 in 40,000 to 1 in 117,000, though this may be an under-estimate as newborn screening in Italy indicates a prevalence of up to 1 in 8,800 newborns (Pieroni et al. 2021; Burlina et al. 2018). As with most enzymatic deficiencies, the degree of enzymatic dysfunction is associated with the age of onset and severity of the clinical course. As Anderson-Fabry disease follows an X-linked pattern of inheritance, it predominantly affects males, with α-galactosidase activity at normal or only modestly reduced levels compared to males, and a corresponding less severe presentation and clinical course.

The classic manifestation of Anderson-Fabry disease results from a severe reduction in α-galactosidase activity (< 1% of normal level), leading to an early onset (< 17 years old) multi-organ involvement, including cardiac features often accompanied by kidney dysfunction, neuropathic pain, and skin and eye abnormalities (Iorio et al. 2024). In contrast, males with more modestly reduced α-galactosidase levels typically have a predominantly cardiac presentation, including heart failure, arrythmia, angina and syncope (Linhart et al. 2007). Unlike most sphingolipidoses, there is minimal direct central nervous system involvement in Anderson-Fabry disease, though cerebral vascular changes and resulting hypoperfusion are a common feature but the factors underlying their manifestation are incompletely understood (Schiffmann and Moore 2006).

Given the multi-system involvement that characterises Anderson-Fabry disease, many previous reports have outlined histopathological observations in multiple organ systems. A consistent observation in histopathological studies of heart, kidney and skin is enlarged cells filled with undegraded storage materials, presumably Gb3, typically alongside fibrosis (Chimenti et al. 2008; Tøndel et al. 2008; Navarro et al. 2010). Relatively few studies have investigated the central nervous system in Anderson-Fabry disease but studies in small numbers of cases have reported ballooned neurons, enlarged axons and prominent glycolipid accumulation in vessel walls (Okeda and Nisihara 2008; de Veber et al. 1992).

α-galactosidase activity is decreased in PD brain tissue, suggesting alterations in Gb3 degradation could occur in α-synucleinopathy though whether these precede α-synuclein aggregation is not clear (Nelson et al. 2018). However, individuals with Anderson-Fabry disease may be at increased risk of developing PD, further suggesting an association between the metabolic dysfunction associated with Anderson-Fabry disease and α-synucleinopathy (Wise et al. 2018). On this basis, a neuropathological study sought to determine whether parkinsonism in Anderson-Fabry disease was associated with Lewy body pathology, identifying pathological changes consistent with PD in a single patient with parkinsonism that were not observed in a case with no clinical history of parkinsonism (Del Tredici et al. 2020). Perhaps surprisingly, α-synuclein accumulation has been identified in the renal glomeruli of Anderson-Fabry disease patients, with in vitro studies indicating that knockout of α-synuclein is more effective than α-galactosidase enzyme replacement therapy at attenuating podocyte degeneration in Anderson-Fabry disease (Braun et al. 2023). Taken together, these findings indicate that α-galactosidase deficiency may contribute to α-synuclein aggregation and that α-synucleinopathy may play a pivotal role in Anderson-Fabry disease, though it remains unclear how deficiency of this degradative pathway contributes to the aggregation of α-synuclein.

In summary, there is growing evidence to indicate α-synuclein aggregation is a feature of Anderson-Fabry disease though, perhaps surprisingly, the strongest evidence to date is for a role in renal dysfunction. It remains unclear whether α-galactosidase dysfunction or the accumulation of Gb3 is responsible for the accumulation of α-synuclein in Anderson-Fabry disease.

## Discussion

There is growing interest in the link between sphingolipid dyshomeostasis and the aetiology of PD and related LBDs, suggesting these should be considered not only proteinopathies, but also as lipidopathies (Flores-León and Outeiro 2025). In addition to genes encoding sphingolipid catabolic enzymes mediating risk of developing Lewy body diseases, there is growing evidence that sphingolipid storage disorders often manifest α-synuclein pathology, even in paediatric populations, suggesting a direct link between sphingolipid dyshomeostasis and the development of α-synuclein pathology. Given the central role ascribed to α-synuclein aggregation in LBDs and the lack of disease-modifying therapies for these conditions, targeting sphingolipid metabolism may be a useful strategy to treat LBD. Recent trials of drugs such as ambroxol, which enhances the activity of glucocerebrosidase, suggest that this approach could be viable for the treatment of PD and DLB (Silveira et al. 2019; Chwiszczuk et al. 2023).

Questions remain about the role that sphingolipids may play in Lewy body diseases. Firstly, it is not clear if variants associated with Lewy body disease are associated with enzymatic loss of function as they are in sphingolipidoses, thus whether such variants lead to reduced enzymatic activity requires elucidation. For example, a recent study indicated that *GALC* variants associated with PD may in fact increase activity of galactocerebrosidase rather than decrease it, as with Krabbe disease (Senkevich et al., 2023b). Several sphingolipid catabolic enzymes implicated in Lewy body disease are primarily expressed in oligodendrocytes and, as a result, their dysfunction typically results in leukodystrophy, such as in Krabbe disease and MLD. However, Lewy body diseases primarily affect the grey matter, with minimal or absent white matter pathology. Our previous studies of Krabbe disease and MLD identified seed-competent α-synuclein pathology in grey matter regions, with white matter not demonstrating a significant reaction on seeded aggregation assay, suggesting that white matter accumulation of particular sphingolipids paradoxically leads to grey matter α-synucleinopathy (Hatton et al. 2022; Ghanem et al. 2024). The mechanism underlying white matter sphingolipid dyshomeostasis influencing grey matter proteinopathy requires further study.

A further question is whether LBDs are in fact late-onset sphingolipidoses. Previous studies have suggested dysregulation of particular sphingolipid species in PD, perhaps indicating that Lewy body diseases such as PD share similarities with sphingolipidoses (Lin et al. 2019). However, the sub-cellular compartment in which sphingolipids are accumulating is unknown, and thus it is unclear whether this accumulation is the result of impaired lysosomal degradation of sphingolipid substrates due to enzymatic deficiency. As noted above, the factors mediating α-synuclein aggregation in the context of sphingolipidoses are unknown, and could be the result of impaired enzyme activity, accumulation of substrates that can cross-seed α-synuclein aggregation, or a combination of both (Erskine et al. 2021). Nevertheless, sphingolipid dyshomeostasis is an attractive hypothesis to explain the diversity of neuropathological changes observed in Lewy body diseases as their accumulation is known to lead to a number of pathological changes thought to be important in Lewy body diseases, such as inflammation, mitochondrial dysfunction and impaired autophagy (Abed Rabbo et al. 2021; Knupp et al. 2017). Therefore, accumulation of sphingolipids could explain the range of pathological features associated with Lewy body diseases, but further studies need to characterise sphingolipid abundance in Lewy body disease brain tissue and the subcellular location in which they are accumulated.

A further outstanding question is whether the accumulation of α-synuclein into seed-competent conformers in sphingolipidoses such as Gaucher disease, Krabbe disease, MLD, and Anderson-Fabry disease, means these disorders should be classed as α-synucleinopathies. The definition of α-synucleinopathy is not clear and could range from any disorder in which α-synuclein accumulates to clear demonstration of a role for α-synuclein in the disease process. In the case of Anderson-Fabry disease, α-synuclein seems to mediate pathological degeneration in kidney, but knockout of α-synuclein only improves histopathology but does not lead to phenotypic improvement (Braun et al. 2023). A study in the mouse model of Krabbe disease, the twitcher model, reported only modest improvements in survival but significant improvement in phenotype with *Snca* knockout, suggesting α-synuclein plays a role in the pathological progression of Krabbe disease (Abdelkarim et al. 2018). Further studies are required to elucidate the role of α-synuclein in the pathological progression of sphingolipidoses, not least as these may help determine whether anti-α-synuclein therapies in development may be beneficial in treating sphingolipidoses, which remain broadly untreatable and are fatal, often in early life.

In summary, α-synuclein pathology is increasingly recognised as a feature of sphingolipidoses that are mechanistically linked to LBDs. Further studies are warranted to better understand this association and the extent to which it can be therapeutically exploited for the attenuation of both LBDs and sphingolipidoses.
